# Facilitating gynecological examination and long acting reversible contraception for women with substance use disorder: a prospective cohort study

**DOI:** 10.1186/s12905-025-03794-0

**Published:** 2025-05-24

**Authors:** Trine Finanger, Ragnhild Bergene Skråstad, Catherine Appleton, Cecilie Therese Hagemann

**Affiliations:** 1 Clinic of Substance Use and Addiction Medicine, St. Olavs hospital, Trondheim University Hospital, Trondheim, Norway; 2 Department of Clinical and Molecular Medicine, Norwegian University of Science and Technology, NTNU, Trondheim, Norway; 3 Department of Clinical Pharmacology, St. Olavs hospital, Trondheim University Hospital, Trondheim, Norway; 4 Centre for Research and Education in Security, Prisons and Forensic Psychiatry, St. Olavs hospital, Trondheim University Hospital, Trondheim, Norway; 5 Department of Mental Health, Norwegian University of Science and Technology – NTNU, Trondheim, Norway; 6 Department of Obstetrics and Gynecology, St. Olavs hospital, Trondheim University Hospital, Trondheim, Norway

**Keywords:** Oral contraceptives, Intrauterine device, LARC, Substance use disorder, Opioid substitution therapy, ThinPrep Pap Test, Cervical screening, Sexual assault

## Abstract

**Background:**

Women with substance use disorder (SUD) face a higher risk of sexual assault, abuse, cervical premalignant lesions, and unplanned pregnancy compared to the general female population. To meet the specific needs of this vulnerable group, we established a comprehensive gynecological service. The study aimed to describe the characteristics of women with SUD who accessed these services. Furthermore, we evaluated their cervical cytology and sexually transmitted infections (STIs) test results, preferred contraceptive methods, and the overall acceptability of the service.

**Methods:**

We enrolled 48 women undergoing outpatient or inpatient treatment for SUD in the South-Trøndelag region of Norway in the study. Each women received a comprehensive gynecological anamnesis, including a history of sexual assault, followed by a gynecological examination at the outpatient clinic of the Department of Obstetrics and Gynecology. The examination included ThinPrep Tests and microbiological swabs. They were also offered contraception options, with an emphasis on Long Acting Reversible Contraceptives (LARC), at no cost. Participants provided feedback on the service immediately after their consultation and again six months later.

**Results:**

Nearly two-thirds (63%) of the women with a mean age 31.8, reported a history of sexual assault. One in three (33%) had cervical cytology or human pappilomavirus (HPV) test results necessitating treatment or follow-up, though no cases of sexually transmitted infections were found. Two thirds (66%) of eligible women chose LARC as their contraceptive method. Among the 23 women reached for a follow-up phone call six months later, all expressed high level of satisfaction with the service and indicated they would recommend it to other women in similar situations.

**Conclusions:**

An integrated, specialized gynecological service that provides cervical testing and contraceptive counselling can effectively reach women receiving outpatient or inpatient treatment for SUD, who typically fail to follow-up routine women’s health screening programmes. If implemented, this approach has the potential to reduce unplanned pregnancies and improve early detection of cervical pathology in this vulnerable population.

## Background

Substance use disorder (SUD) among women of reproductive age pose a significant public health concern due to its potential impact on pregnancy and long-term maternal and child health. The burden of disease from alcohol and drug use varies across countries and gender, and although men still have higher rates of substance use disorders, the gender gap is narrowing [1, 2]. In the United States, the lifetime prevalence of alcohol and drug use disorders among women is estimated at 19.5% and 7.1%, respectively [3]. Notably, the age group with the highest rates of SUD overlaps with the most fertile years [4, 5].

Women under the influence of drugs are more likely to engage in risky sexual behaviours, experience sexual violence, and endure sexual abuse [6, 7]. Women with SUD who have experienced sexual abuse face a higher risk of developing lifelong post-traumatic stress disorder, anxiety, and chronic pelvic pain [8]. This underscores the critical importance of providing tailored, comprehensive, and readily accessible gynecological health services to effectively reach and support this vulnerable population.

Due to their engagement in risky sexual behaviours, women with SUD face an increased risk of exposure to Human Papillomavirus (HPV), a primary cause of over 95% of cervical cancers [9–11]. Despite their heightened risk of developing cervical dysplasia, compared to the general female population [10, 12], women with SUD have lower attendance rate in screening programmes designed for the early detection of cervical cancer [9, 13, 14].

Another consequence of risky sexual behaviours is that it contributes to an increased risk of contracting and transmitting sexually transmitted infections (STI) [15, 16].

For various reasons, women with SUD utilise contraception less frequently [17]. Approximately 80% of their pregnancies are unplanned, a stark contrast to the 50% rate observed in the general female population [12, 18–22]. As a consequence, women with SUD often find themselves in situations where they will seek a legal abortion or are not secured custody of their children [20]. Moreover, exposure to various potentially teratogenic substances of abuse due to maternal SUD is complex and uncertain, with the potential to result in structural anomalies and permanent brain damage to the foetus [23–25]. In addition, the use of opioids alone, or in combination with polydrug abuse, can lead to withdrawal and adaptation disorder in the newborn child [26, 27]. Hence, it becomes imperative to facilitate family planning and provide safe contraception options in order to help patients with SUD have healthy families and healthy pregnancies as a future possibility. This is where long-acting reversible contraception (LARC), such as intrauterine devices (IUD) or contraceptive implants, comes into play. They stand as the preferred choice for all women due to their reliability and low risk profile [19, 28]. In alignment with this, the Norwegian Directorate of Health strongly recommends the utilization of LARC in its guidelines for women with SUD or in opioid substitution therapy (OST) to reduce the incidence of unplanned pregnancies until individuals attain a drug-free living situation [29]. The IUD is a safe and cost-effective contraception option, with high compliance, user satisfaction and minimal side effects [30]. Its use minimises user failures, and fertility returns quickly after removal [19, 31, 32]. This latter is particularly valuable for women planning pregnancies at a later, more suitable time.

Whilst there have been several studies addressing this patient population's contraception desires and needs, further research is needed to assess the implementation and impact of specialized services in clinical practice [19, 20, 33–38]. According to a literature review by Black et al. the evidence suggests that women with SUD face an increased risk of unintended pregnancy. Despite this elevated risk, they typically have limited access to effective contraception, particularly LARC, due to factors such as cost barriers, multiple referrals, stigma, and concerns about child protection. Studies that assess the benefits of integrating contraception services into drug treatment programmes are small, and the review concludes that further research is needed to identify effective and acceptable models [38]. To address the unique needs of this subpopulation, we established a novel comprehensive, specialized service for women with SUD at St. Olavs hospital, Trondheim University Hospital, Norway, in 2017. This location was chosen because it is where the study lead and research group are based. The service provided free gynecological examination by a trained gynecologist, including cervical cytology screening, microbiology testing and access to free contraceptives. While patients were free to choose their preferred contraceptive method, healthcare workers and consulting gynecologist emphasized the use of LARC.

The aim of the current study was to describe our comprehensive gynecological service provided and describe the women with SUD who accessed this service. Additionally, we evaluated their cervical cytology and STI results, preferred contraceptive methods, and acceptability of the service.

## Materials and methods

### Design and setting

The study was conducted as a prospective cohort study, involving women aged 18 years and above with a history of current or previous SUD and/or those undergoing OST, who received outpatient or inpatient treatment at a primary or secondary health care centre within the area of Trondheim, Norway.

### Recruitment and study inclusion procedure

Beginning on 1 st September 2017, we offered women undergoing inpatient or outpatient treatment at the Clinic of Substance Use and Addiction Medicine, St. Olavs hospital, or at similar facilities within Trondheim municipality, a free gynecological examination and access to free contraception. The physician excluded patients who appeared to be under the influence of substances or otherwise incapable of providing informed consent. The service was initially introduced as a pilot project. Originally planned to span across one year, the study period commenced on 1st September 2019. However, due to the Covid-19 pandemic, the gynecological outpatient clinic experienced prolonged closures. Consequently, the inclusion period was extended until 1st May 2021.

Health care workers at drug rehabilitation and outpatient clinics led the recruitment for this service. They informed women with SUD, including those in OST, about the service, and scheduled appointments for those who were interested. In Norway, gynecological examination and LARC are typically provided by general practitioners (GPs). However, for women with SUD, scheduling and affording these appointments and contraceptives can be challenging. This service was specifically designed for those unable to access these consultations through their GP. To reach as many candidates as possible, staff at various healthcare institutions informed women they believed would benefit from the service. Since the recruitment was done pragmatically, it was not possible to track how many women were considered unsuitable or how many declined the offer. These consultations were offered weekly at the outpatient clinic within the Department of Obstetrics and Gynecology at St. Olavs hospital, Trondheim University Hospital. Most participants, but not all, were accompanied by a staff member from the referring clinic for support and assistance in locating the clinic. Upon arrival at the gynecological outpatient clinic, before the examination took place, the women were provided with both verbal and written information regarding their option to participate in the study. The women were assured that their medical treatment would remain unchanged regardless of their participation in the study, and no additional incentives were offered for taking part. All participants recruited for the study were provided with an informed consent form, which they read and signed. The form was available in written format, and upon request, it was also explained orally.

### The medical examination and laboratory testing

The medical examination consisted of a comprehensive assessment, encompassing a thorough review of the woman’s medical history and her existing health concerns including substance use, socio-demographic and gynecological history at inclusion. Additionally, the woman was informed of the planned gynecological procedure. These examinations were conducted by a specialist in obstetrics and gynecology (the first author of the study), with a nurse from the gynecological outpatient clinic always present. Both the consultation and the LARC were provided free of charge to the woman. Oral contraceptives were also offered at no cost during the inpatient stay and prescribed upon discharge. Since blood tests are not typically part of a standard gynecological examination, routine testing for HIV, Syphilis or Hepatitis C was not performed. However, most of the patients were inpatients, where these tests are part of the standard admission procedure. The collection of cervical cytology was in accordance with the guidelines of the Norwegian screening programme for cervical cancer, performed using a broom shaped brush, which was immersed in a ThinPrep liquid, facilitating testing for both Pap smears and HPV [39]. Cytology was used for women aged 25–33 years old, and HPV DNA-testing was conducted for women aged 34–69 years old. In cases where macroscopic abnormalities were detected during the examination, cytology was always performed. The HPV test utilized during this period was the Cobas 4800 HPV test (Roche Molecular Systems, Pleasanton, USA) which examined the high-risk HPV L1 gene. The test results included genotyping for HPV 16 and HPV 18, as well as pooled results for 12 other high-risk types (31, 33, 35, 39, 45, 51, 52, 56, 58, 59, 66 and 68).

Vaginal swabs for potential sexually transmitted infections (STIs), such as Chlamydia Trachomatis (CT) or Neisseria Gonorrhea (NG) were also conducted. Between May 2019 and September 2020, CT and NG real-time PCR (polymerase chain reaction) were administered using the FTD Urethritis basic kit from Fast Track Diagnostics (Luxembourg). From October 2020 to September 2021, the tests were changed to the STI AMP Kit, Alinity m, manufactured by Abbott Diagnostics (Wiesbaden, Germany). Additional microbiological tests were performed for Trichomonas Vaginalis, bacterial vaginosis, and Candida Albicans.

The ThinPrep Pap Tests and microbiological swabs were sent for analysis to the Department of Pathology and the Department of Medical microbiology, respectively, both located at St. Olavs hospital.

Transvaginal ultrasound examination and the insertion of IUD were performed when deemed appropriate. Women who had decided to receive an IUD before the consultation were advised to take prophylactic analgesics (1g paracetamol and 600mg ibuprofen) one hour before the procedure. For those whose IUD insertion was not pre-planned, painkillers were administered immediately after the procedure. While not routinely offered, a local anaesthetic containing prilocaine and felypressin was provided as a paracervical block for those who requested or required it prior to IUD insertion. The same anaesthetic was applied to the upper arm before implant insertion. No sedatives were administered before or during the examination.

Subsequently, all women were informed about any pathological test results via phone, and they also received a formal letter (with a copy sent to their GP). Information regarding the necessity for any follow-up, was provided, and further referrals arranged as required.

### Women’s reported outcomes, immediate and at six-months follow-up

Immediately following the consultation, participants were invited to complete a questionnaire survey. This survey consisted of 10 items designed to assess their experience during the recent gynecological examination. Each item provided three possible response options. The questions covered various aspects, including, amongst others, the women’s perceptions of the examination process, the testing procedures, the ultrasound examination, and, if relevant, their experience of the insertion of an IUD (Table 3).

Six months after the initial examination, the women were recontacted to engage in a follow-up phone interview. This interview consisted of 12 questions, with response options limited to mainly ‘yes’ and ‘no’. The questions encompassed various aspects, including whether they were still using the contraceptive method provided during the examination, their level of satisfaction with the contraceptive, any reported side effects, and whether they had experienced pregnancy in the last six months. In addition, participants were asked to express their level of satisfaction with the specialized gynecological service they had attended (Table 4).

### Data collection

Information regarding the participants´ medical history and clinical data was documented using a paper-based registration form, which was completed by the gynecologist immediately after the consultation. Additionally, laboratory test results and relevant information were subsequently transferred onto the same registration form. All data, including the immediate self-reported outcomes from the women and the information gathered during the follow-up interviews, were de-identified (names were replaced by numbers) and consolidated into an SPSS datasheet and merged for subsequent analysis. All data were securely stored in two separate research study file locations – one for the key code and the other for the de-identified data – both provided by the hospital’s Security Department.

### Statistics

Descriptive statistics were conducted, and quantitative data were analyzed using SPSS Version 25.0. (Armonk, NY, USA).

## Results

### Substance use, socio-demographic and gynecological data

**Fig. 1:**
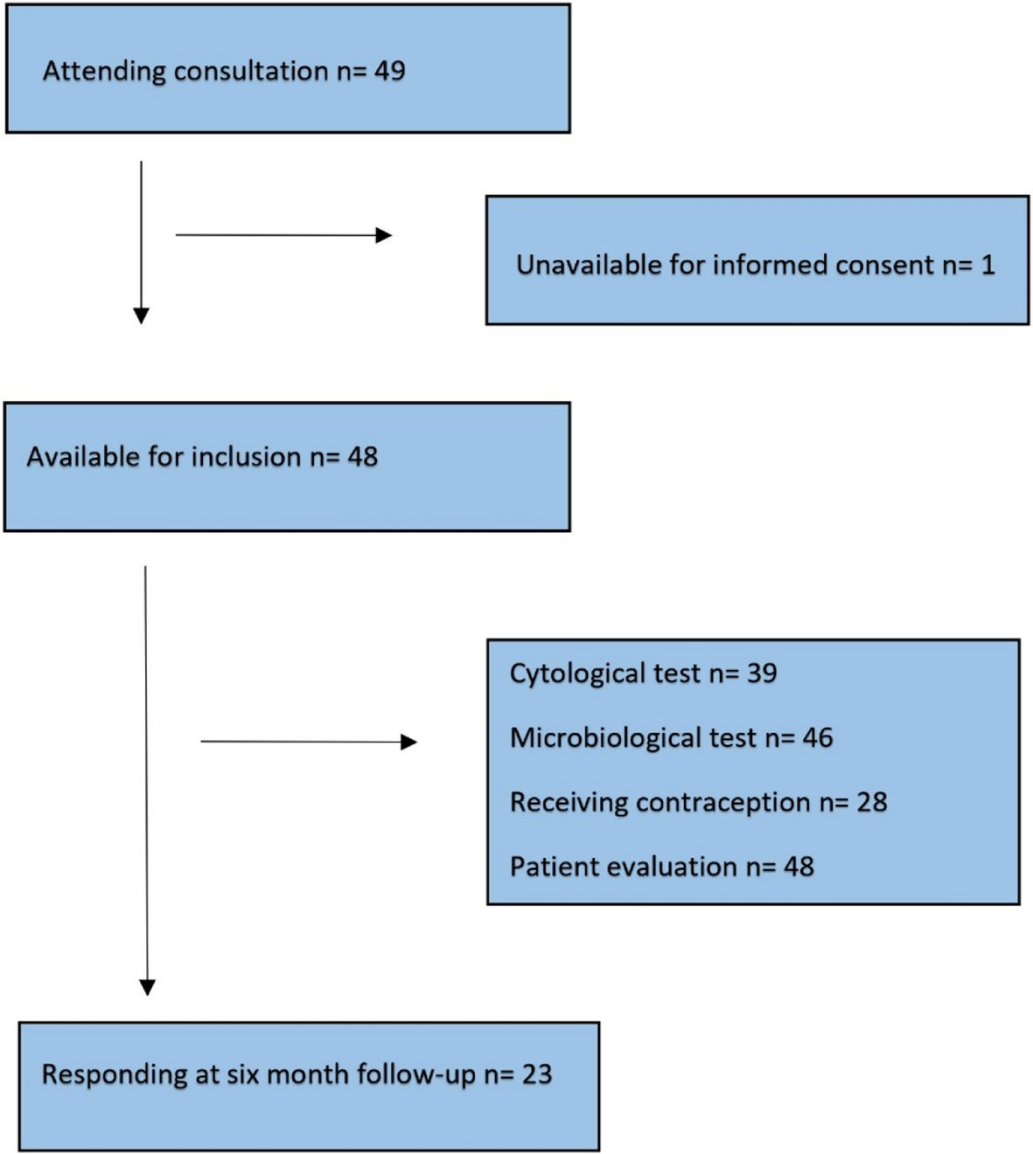
Summary chart of the study

Among the 49 women attending the service in the study period, one woman was considered incapable of providing full and informed consent (Fig. 1).

A total of 25 women reported intravenous drug abuse as part of their substance use history. The most commonly abused drugs were stimulants (amphetamine, cocaine), reported by 19 women, followed by opioids, alcohol, cannabis, and benzodiazepines in descending order (Table 1). The average age at attendance was 31.8 years, and among the 21 women who had children of relevant age, 11 were actively caring for their own children. In total, 46 of the 48 women had previously tried some form of contraception, including oral contraceptives, patches, LARCs, or condoms. Four of them had somatic conditions that contraindicated the use of oral contraceptives and/or oestrogen (such as protein S deficiency, bulimia and gastric bypass). However, of those using oral contraceptives, none were on medications or suffered from conditions that contraindicated their use. A total of 27 women opted to use contraceptives; further details are provided below. In addition, among 34 women who had ever been pregnant, 24 (71%) reported having had legal abortions, and of a total of 46 women, 29 (63%) reported a history of sexual assault.

**Table 1:**
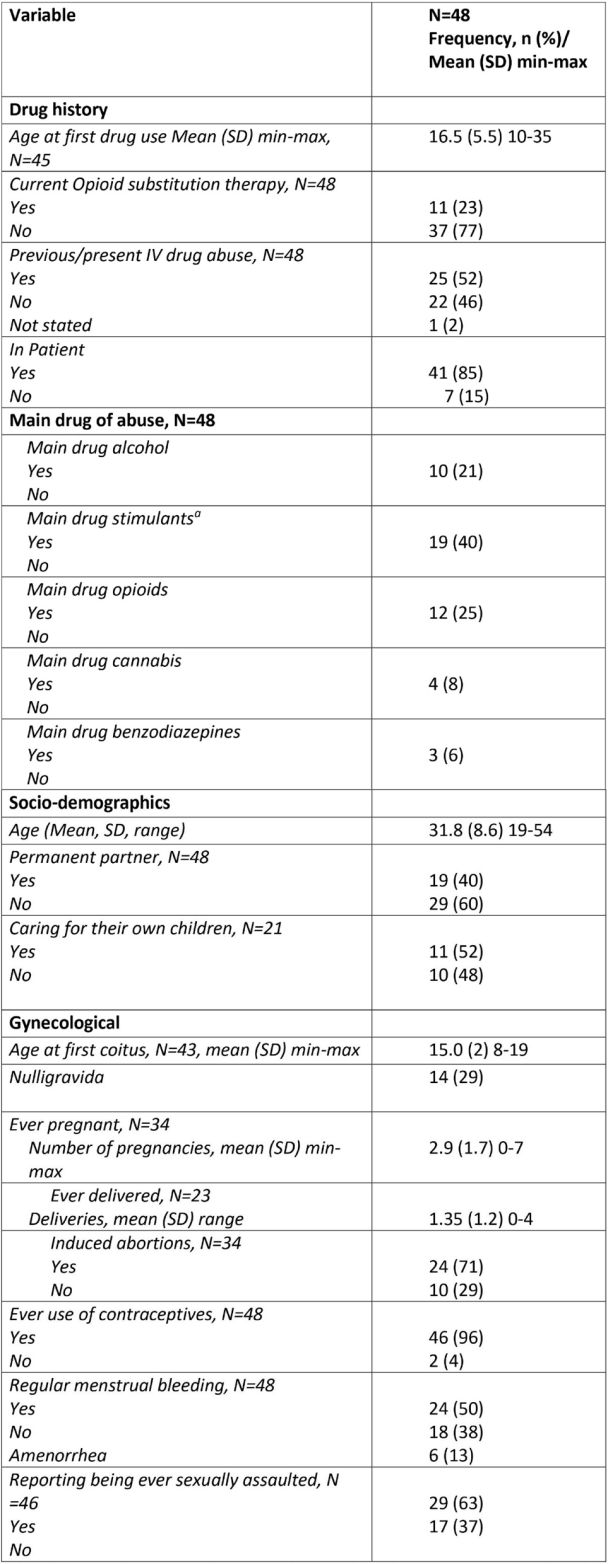
Demographic characteristics, gynecological history and substance use among the 48 women who attended the gynecological consultation

^a^ Central nervous stimulants include, among others, amphetamine, metamphetamin and cocaine.

### Laboratory results

Nine participants in the study did not undergo cytology and HPV testing. This exclusion was due to various reasons: four of them were considered too young for inclusion in the Norwegian cervical screening programme (which is recommended from the age of 25), one had already undergone cervical removal surgery and four reported recent testing. In total, 39 women underwent cervical cytology and/or HPV tests, and among them, 13 showed pathological results (Table 2). Five women with results indicating Atypical Squamous Epithelial Cells, Cannot Exclude High Grade Squamous Intra-epithelial Lesion ASC-H or another high-risk outcome subsequently underwent cervical biopsies, which revealed moderate to severe dysplasia. All of these women received surgical conization as treatment. An additional eight women had lower-grade results and were advised to undergo re-testing in accordance with screening guidelines within 6–24 months [40].

**Table 2:**
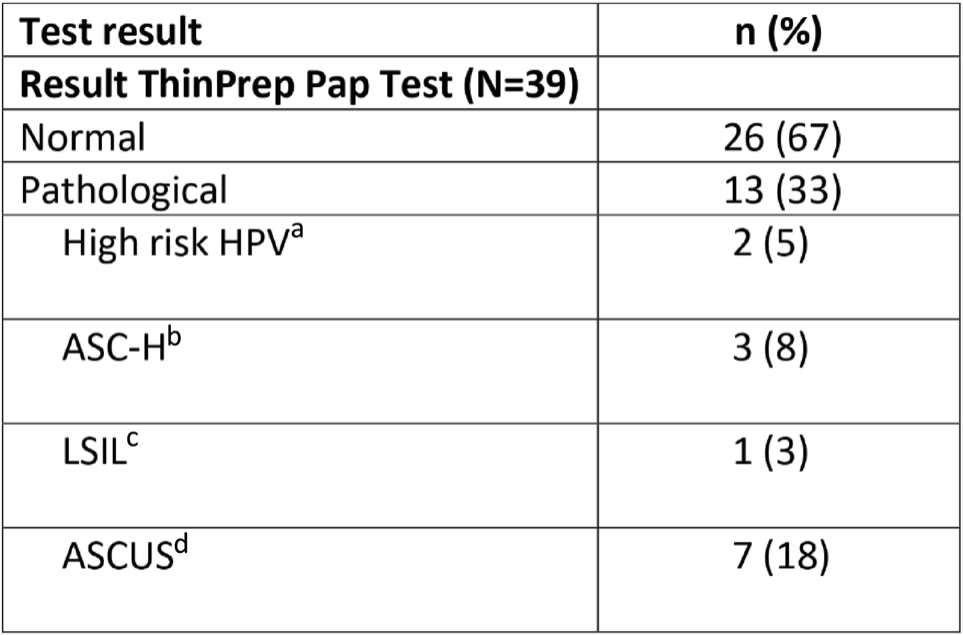
Cervical cytology and HPV results in women with Substance Use Disorder attending the gynecological service

Five women had two pathological test results (LSIL and AGUS plus High risk HPV)

^a^ Human Papilloma Virus 16, 18 or other high risk virus

^b^ Atypical Squamous Cells, Cannot Exclude High Grade Squamous Intra-epithelial Lesion

^c^ Low- grade Squamous Intraepithelial Lesion

^d^ Atypical Squamous Cells of Undetermined Significance

None of the 48 women tested positive for STIs, and none exhibited signs of Pelvic Inflammatory Disease during clinical examination. However, Candida Albicans and/or Gardnerella Vaginalis were detected in eight women, and appropriate treatment was administered in response to any symptoms.

### Gynecological and contraceptive counselling

A total of 21 women received no contraception during the consultation. Among these, one was postmenopausal, one had a hysterectomy, one had tubal ligation and seven were already using contraceptives (two had an implant, three had an IUD, one had depo contraceptive injection (Medroxyprogesteronacetat), and one was taking an oral contraceptive). A total of 11 (23%) chose not to use contraceptives.

Of the 27 women who desired contraception, 23 (85%) opted for the IUD as their preferred choice. Among this group, all but one woman requested a hormonal IUD. Prior to the IUD-insertion, six women received local anaesthesia. Additionally, two women chose oral contraceptives and two opted for implant.

Furthermore, a vaginal ultrasound was conducted for all women, and in one instance, an ovarian cyst was detected, necessitating follow-up.

### Patient evaluation

All 48 women completed the post-examination questionnaire immediately following the exam (Table 3). When it came to IUD insertion, four of the 23 women who underwent the procedure reported it as “worse than expected”, none of these four received local anaesthesia.

**Table 3:**
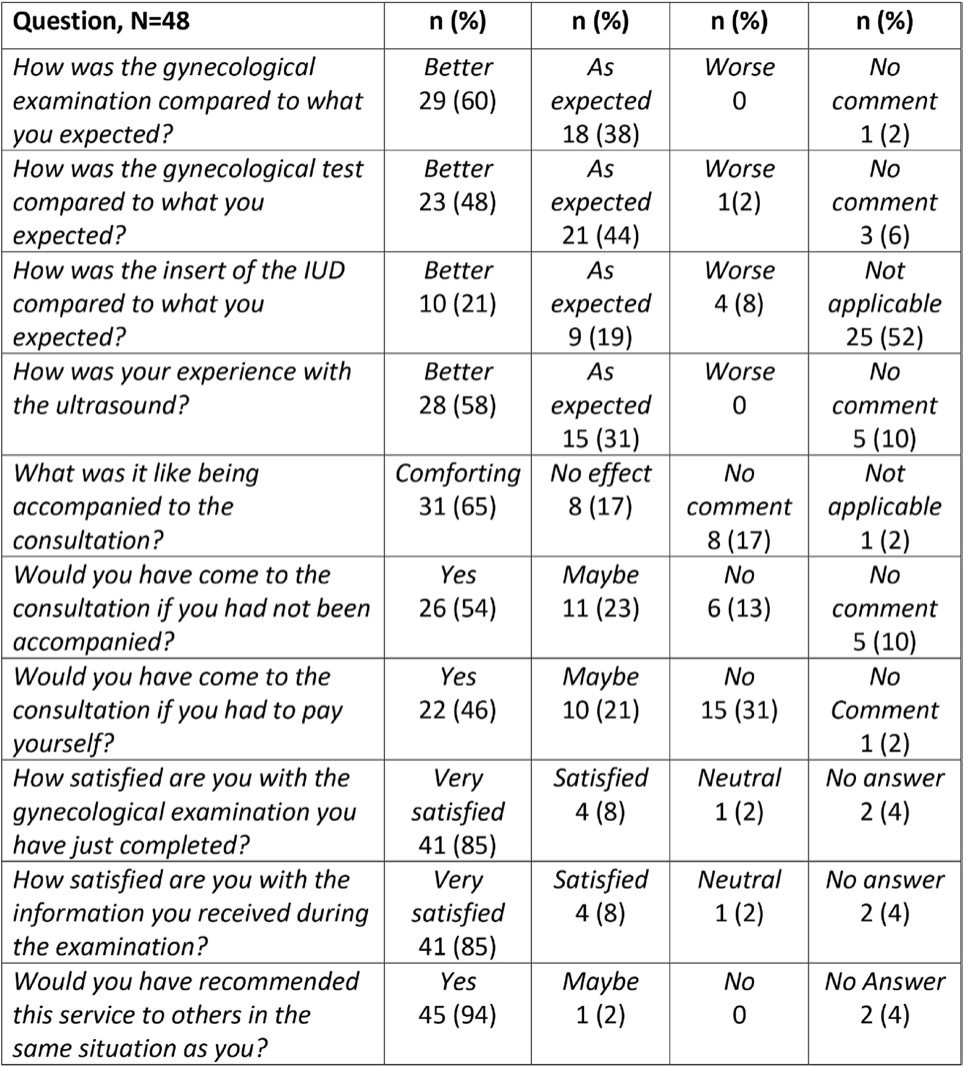
Self-report evaluation of the 48 women with substance use disorder immediately following the gynecological examination

Six months after the gynecological consultation, we successfully established follow-up contact with 23 out of the 48 women (Table 4). Among these individuals, 11 had received contraception at the time of the examination, and 9 out of those 11 women reported still utilising the contraception provided. Among the two women who had discontinued the contraceptive, both had initially used IUDs. One of them had replaced the IUD with an implant, while the other participant had the IUD removed due to irregular bleeding and had chosen not to use any contraception at present. Notably, during the six-month follow-up period, none of the 23 women reported experiencing a pregnancy.

**Table 4:**
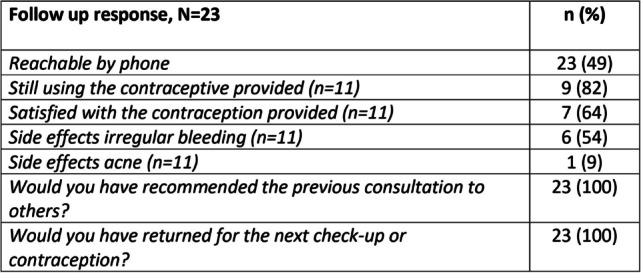
Follow-up report of women with substance use disorder six months after the gynecological examination

## Discussion

Nearly two thirds of the women in our study had experienced sexual assault, one in three received a pathological cervical cytology test result necessitating treatment or follow-up, and two thirds of the women in need of contraception expressed a preference for LARC. Of major importance, however, was the high level of satisfaction among the women provided the service, as almost all of them expressed that they would recommend and revisit the service if necessary.

The importance of contraception in this study population is highlighted by our data, which revealed a high rate of previous legal abortions −71% (24 out of 34). Additionally, only half of the 21 women with children in the relevant age group had custody of their biological children. It is well known that mothers with SUD are more likely to have their children placed in foster care and are at higher risk of permanently losing their parental rights. Factors that increase the likelihood of children being placed in out-of-home care include unplanned pregnancies, having a large number of children, and a history of previous loss of parental responsibility [41–43]. Consequently, ensuring planned pregnancies, especially until they have recovered from SUD, by providing safe and cost-free reversible contraception, could significantly aid in retaining custody of their children.

Our findings that more than 60% reported incidents of sexual assault is consistent with previous international studies that have reported similar statistics [44, 45]. Women who have been subjected to sexual abuse face an increased risk of lifelong post-traumatic stress disorder, anxiety, and chronic pelvic pain [8]. Consequently, it is imperative for gynecologists to possess the professional competence necessary to effectively address these women’s specific needs. A history of sexual abuse can significantly impact a woman`s mental and physical health, making it challenging for her to access gynecological services. Understanding trauma-informed care is essential for conducting sensitive and compassionate gynecological examinations, with the goal of preventing re-traumatization. This approach plays a key role in influencing a woman`s willingness to participate, her sense of safety during the examination, and her overall experience during the consultation.

When the facilities enabled examinations for this patient population, one in three of the 39 women who underwent testing had a pathological cervical test result that required treatment or follow-up. Among these, five of the 13 women with abnormal cervical test results underwent surgical conization. Given the high incidence, and the potential for early detection and treatment, cervical pathology screening programmes have been implemented in the national health care programme for early detection and prevention of mortality in 139 out of 202 countries [46, 47]. Subpopulations, such as women with SUD, experience a significantly higher incidence of cervical HPV infection and precancerous lesions, however, they tend to have lower attendance rates at the cervical screening programme [10, 48–50]. Therefore, it is recommended to provide easily accessible cervical cancer screening facilities for women with SUD [48, 51].

Since our study offered gynecological examinations while the women were in a treatment setting for substance use, it is likely that we included women with a more severe SUD, which might lead to an even higher likelihood of non-compliance with the screening programmes. In our study, 33.1% of patients required follow-up (such as biopsies) and 12.8% needed conization due to moderate or severe dysplasia. These rates are significantly higher compared to the national Norwegian screening programme, where only 6.1% require follow-up, and 3.6% undergo conization [52]. Although a small sample size, our results together with findings in similar studies, highlights the unmet need for a facilitated service tailored this vulnerable subpopulation. However, it is worth noting that previous research has shown that once pathology is detected, women with SUD exhibit similar adherence to complex treatment plans, resulting in comparable cancer specific outcomes when compared to women without SUD [53]. In retrospect, it is worth considering whether HPV vaccination should have been offered during the consultation or upon admission to a drug treatment facility, similar to the hepatitis B vaccination. Given that this patient group has a significantly higher rate of pathological cervical cytology findings and a much lower likelihood of undergoing the ThinPrep Test, offering HPV vaccination could be an important preventive measure.

In contrast to previous studies where STIs and SUD were recognized as comorbid conditions [54], our study did not detect any cases of STIs. One possible reason for this discrepancy is that the inclusion period of our study coincided with the Covid-19 pandemic, during which there was a general decrease in the frequency of STIs [55]. Probably more importantly, the women included in our study were enrolled in drug rehabilitation programmes, where urinary tests for STIs (as well as blood serology) are routinely conducted upon admission to the ward, and antibiotics would have been initiated if positive STI test.

A primary aim of the study was to provide contraception, with an emphasis for LARC, and more than half of the eligible women in our study opted for this method. Considering that some of the women already had contraception, had undergone surgery that made contraception unnecessary, or were postmenopausal, the actual contraceptive rate among the study population reached as high as 76%. The provision of no-cost contraception, along with easy access and information about the effectiveness and safety of LARC, has also previously shown significant impact. In the CHOICE study, which involved nearly 10 000 women and adolescents in a general population in the St. Louis region in the United States, this approach led to a remarkable increase, from 5% to nearly 75% of women choosing LARC. The intervention resulted in a statistically significant reduction in abortion rates and a high rate of continuation and satisfaction with the contraception provided [32, 56]. Previous research has indicated that women with SUD may initially exhibit a lower interest in LARCs [36]. Nevertheless, once administered, they seem to adhere to LARC methods in a similar manner to women without SUD [57]. A study conducted in Baltimore, USA, specifically to assess the attitudes toward family planning among women with SUD confirmed their satisfaction with receiving contraceptive education and services while undergoing SUD treatment. Furthermore, they expressed a preference for integrated services [58]. This sounds reasonable since there has been disparities between prenatal intentions and the actual receipt of LARC postpartum among pregnant women with SUD [33]. Consequently, delays in securing appointments and logistical challenges can pose difficulties for women with SUD in attending referral appointments for IUD insertion, which in turn can impact their choice of contraception and abortion rates [58]. Therefore, offering comprehensive knowledge, no-cost services, co-location, and the initiation of gynecological and reproductive health services integrated in (on the same day as) SUD treatment programmes may increase the use of LARC [56, 58].

The most encouraging finding from this study was the high level of patient satisfaction post-consultation. Since only half of the participants could be reached by phone after six months, no firm conclusion can be drawn due to the small study population. However, we have no reason to believe that their lack of response was related to concerns about the study, as the contact number was unidentifiable. Among the women who were successfully reached, all reported being very satisfied with the service. They expressed their willingness to recommend it to others and return for a check-up when needed, despite approximately half of them reporting side effects like irregular bleeding or acne (Table 4). Importantly, the follow-up rate of 50% highlights the significance of helping women access healthcare when they are in a relatively stable clinical condition and available for treatment, for which our service was well-designed. Women with SUD are often perceived as too vulnerable, unable or unwilling to provide informed consent, which can result in their exclusion from research, even when the research is highly relevant to their needs. By conducting our study within a treatment setting and reaching the women while they were sober and actively engaged in targeted treatment, we were able to obtain fully informed consent. Doubtless, this study is notable for its comprehensive approach, which involved establishing a complete gynecological service for these women, and capturing their first-hand experiences.

The study does have certain limitations. First, we do not know how many women were offered and accepted the service, making it unclear whether our results reflect a representative sample. This uncertainty applies to both the initial examination and the six-month follow-up. Second, we lack information on whether women with abnormal cytology results followed-up with further examination and treatment. Additionally, the six-month follow-up period – during which only half of the participants were reached—may have been too short to capture the full progression of cervical cytology changes and assess contraceptive continuation. Furthermore, the evaluation of the examination and the responses during phone calls could have been influenced by the interviewer. However, it is important to acknowledge that such potential biases are inherent in all clinical studies and may impact the results to some extent.

## Conclusion

This study highlights the unique gynecological needs of women with SUD, revealing several key findings. Women with SUD often have a history of sexual abuse and face a higher risk of cervical pathology compared to the general female population. Additionally, when offered, they tend to prefer LARC as a contraceptive method. An integrated and targeted gynecological service that includes cervical testing and contraceptive counselling is highly valued by these women. Such services offer individual health and socio-economic benefits by preventing unplanned pregnancies and enabling early detection of cervical pathology. We hope this study inspires the establishment of similar facilities in other regions and countries, enabling further data collection and research on the health outcomes of this vulnerable group of women. Undoubtedly, challenges such as resource limitation and political will may arise, depending on how governments prioritize and support women’s health.

## Data Availability

The datasets used and/or analyzed during the current study are available from the corresponding author on reasonable request.

## Abbreviations

LARC: Long-acting reversible contraception
IUD: Intrauterine device
OST: Opioid substitution therapy
SUD: Substance use disorder
STI: Sexually transmitted infection
HPV: Human papillomavirus
GP: General Practitioner
CT: Chlamydia trachomatis
NG: Neisseria gonorrhoeae
